# Observations on Small-Pox, as It Has Occurred in London in 1825

**Published:** 1826-02

**Authors:** G. Gregory

**Affiliations:** Physician to the Small-Pox and Vaccination Hospital.


### Art. VII.—*Observations on Small-Pox, as it has occurred in London in* 1825. By G. Gregory, M.D. Physician to the Small-Pox and Vaccination Hospital.

It is now three years since I had the honour of submitting to the Medical and Chirurgical Society of London, some "Cursory Remarks on Small-Pox, as it occurs subsequent to Vaccination." The favourable manner in which that paper was received by the public, induces me to think that a continuation of the same inquiry, founded on the results of more recent experience, may not be unacceptable. The circumstances of the times are peculiarly favourable to such an investigation. It is well known to all who have been practising in London, that small-pox has been met with very generally during the last year. With the cold of December, the prevalence of smallpox has diminished, and we are now at liberty calmly and dispassionately to look back upon the events which have passed before us, and to form from them an estimate of the degree of security from this loathsome disease which the world enjoys from the discovery of Jenner. It is to this point, mainly, that the observations which I have now to offer are directed. To enlarge upon the importance of this object of inquiry, would surely be superfluous. Whether we view it in a pathological or in a national point of view,—whether we consider it as affecting ourselves or our patients,—we shall find it equally deserving of our most serious attention.

That small-pox has been decidedly *epidemic* in London during the year 1825, may be deduced as well from public as from private records. The admissions into the Small-Pox Hospital have been greater this year than in any since 1796, a year of such general and fatal small-pox, that 3549 persons are reported in the Bills of Mortality to have died of it; very nearly the largest number recorded since the commencement of the Bills of Mortality in 1592.* The annual admissions into the Small-Pox Hospital have only been exceeded on two other occasions during the course of the last half-century,—viz. in the years 1777 and 1781, when the deaths, by the Bills of Mortality, were 2567 and 3500, being about double the average numbers.

Finding thus that the admissions into the Small-Pox Hospital have always proved a fair index of the extent to which the disease has been prevalent in London, it may be inferred that small-pox has been nearly as general in 1825 as in any of the three great epidemics of the preceding century. And now let me solicit attention to the comparative extent of *mortality* in these several years. Had nothing occurred to alter the scale, it is reasonable to suppose that the deaths by small-pox this year, as reported in the Bills of Mortality, (taking into account the increased size of the town,) would have been at least 4000: yet the numbers reported are, in fact, but 1299; an enormous increase, indeed, upon the former year, which was but 725, but falling infinitely short of what would have happened but for the general prevalence of *vaccination*.

In the course of the last ten years, small-pox has been epidemic in various towns and districts of England: as, for instance, in Edinburgh and its neighbourhood, in 1818; at Norwich, in 1819; at Chichester and the neighbourhood, in 1822; at Oxford, in 1824. The disease in those places excited much more attention, and at Norwich, in particular, the havoc caused by it was comparatively much greater than has occurred in London this last year; the reason of which I believe to be this—that the

* The greatest mortality was in 1763, when 3582 persons died of small-pox.

practice of vaccination has been much more general in the metropolis than in the country, and that the opportunities of procuring fresh and good lymph are here necessarily so much greater.

In 1819, Sir G. Blane read a paper to the Medical and Chirurgical Society, showing, among other things, that vaccination had had a most marked influence in diminishing the general mortality by small-pox. He pointed out that, in the fifteen years preceding 1819, the mortality by small-pox, as reported in the Bills of Mortality, had not been more than one-half of what it was in the two like series of years in the middle and latter end of the last century. A table which I have drawn up of the admissions and deaths at the Small-Pox Hospital during the last fifty years, tend exactly to the same point. The admissions during the first twenty-five years of this century are 3743, and the deaths 1118; while the admissions during the last quarter of the eighteenth century, (when vaccination was unknown,) were 7017, and the deaths 2277, somewhat more than double.

I shall now direct my attention more particularly to the circumstances under which small-pox has presented itself at the hospital of which I have charge, during the year 1825; reserving to the last a notice of those cases which have occurred to me in private practice.

During the past year, 419 persons were admitted into the Small-Pox Hospital. 263 took the disease in the natural way, without previous protection; of whom, 107 died. Two had it subsequent to variolous inoculation; of whom, one died;—and 147 had small-pox after real or presumed vaccination; of whom, twelve died. The rate of mortality among those wholly unprotected was unusually high (forty-one per cent.), and proves the epidemic to have been one of exceeding malignity. The greatest mortality occurred during the months of July and August.

Of the 147 persons who had small-pox after real or presumed vaccination, 122 had the disease in some degree modified, or mitigated, and twenty-five passed through it in its natural and unmodified form.

This recital is certainly calculated to diminish, in a painful degree, the reputation which the cow-pox has so long enjoyed; and it is with much regret that I make such a statement. Yet, though the experience of this epidemic may tend to bring down vaccination from that exalted pinnacle of national importance which it has hitherto held, it would be most dangerous and most unscientific to go into the other extreme,—to abandon it because it does not fulfil all that our too-ardent expectations had anticipated, or to consign to reproach a most useful and most admirable discovery. In considering the cases which have this year occurred at the Small-Pox Hospital subsequent to vaccination, the following circumstances demand attention:—

It must be remembered, 1st, that, though they are very numerous, a large proportion of them were exceedingly mild, and devoid of all *kind* and *degree* of danger. In many the disease was hardly to be recognised as small-pox: it was decidedly the *chicken-pox* of Dr. Heberden, and other authors who wrote prior to the discovery of vaccination. 113 of them were discharged, cured, within fourteen days from the period of admission; and thirty of these actually within seven days. In nine only was the convalescence protracted; and this was attributable either to constitutional weakness, or to accompanying disease. Two or three of them took small-pox after typhus, and others had enlarged scrofulous glands or sore legs at the time of their admission. One of them had supervening scarlatina.

It should be remembered, 2dly, that there must always exist a difficulty in ascertaining exactly the reality of the previous vaccination. My rule, throughout the year, was never to exclude any one from this class who could show a scar, or (failing that criterion) who retained a distant recollection of having undergone some kind of protecting process. In many of the unmodified and fatal cases just referred to, the evidence of prior vaccination was very imperfect; but I do not wish to disguise my conviction that, in others, the proofs of vaccination were distinct and undeniable.

In reference to these last cases, it should be considered, 3dly, that small-pox this year proved exceedingly severe to all who were *unprotected;* and it is not an unreasonable supposition that, to the malignity of this particular epidemic, something is to be attributed. Many circumstances conduce to the belief that vaccination may be capable of successfully opposing the contagion of small-pox in its *ordinary* degree of intensity, though it may fail in affording protection against it when in its *highest* degree of virulence. Had the individuals who have this year suffered severely from small-pox after vaccination been attacked by it in former seasons, they might possibly have passed through the disease mildly and safely. A similar period of general and fatal small-pox not having occurred for the last thirty years, may not recur for thirty years to come; and, consequently, a like unfortunate result with that which I now record may not, during that long period, be again witnessed.

4thly. A strong presumption in favour of the positive advantages of vaccination, may be drawn from this important fact, that, in the year 1825, a greater number of persons have been vaccinated at the Small-Pox Hospital than in any year since the discovery of the cow-pox; and I am informed that, at several of the stations of the National Vaccine Establishment, the numbers vaccinated in 1825 nearly double those of 1824. When we reflect that, during the past year, small-pox has found its way into every alley and lane in London, crowded with the densest population, and that whole families of vaccinated persons, of all ages from birth to five-and-twenty, have been every where fully and freely exposed to small-pox contagion, in a state of great intensity, it follows, I think, to demonstration, that the public (for whose benefit we are anxious, and who are, after all, the best judges of the question,) are really satisfied with the degree of protection which *genuine* vaccination, such as may be had in London, gives. 4003 children were vaccinated at the Small-Pox Hospital in 1825; and I am sure that, of them, more than one-half were brought by their parents under the pressure of small-pox at their very doors. Had they not seen vaccinated persons resisting the contagion effectually, they would not have subjected their infants to vaccination. It appears to me most important to bear in mind that, from the year 1806, when vaccination began to be generally practised at the Small-Pox Hospital, to the present day, the numbers have been steadily and rapidly augmenting. Dividing the last twenty years into four periods of five years each, the numbers have been 7004, 9339, 13,348, and 16,666. We may, perhaps, have heard of a dozen or twenty families in whom small-pox subsequent to vaccination, performed at our establishment, has occurred; but none of these persons have died, and few have been confined to their beds more than four or five days. Nay, more,—manyof the parents of such children, notwithstanding this occurrence, have still brought their infants for vaccination.

The impression left upon my mind by the events of this very interesting year has been, that vaccination has the strongest claims upon the confidence of medical men, and on the national support; and that nothing further is to be desired, in the way of protection against small-pox, than a stricter attention, on the part of practitioners in the country, to the diffusion of good and genuine vaccine lymph, and perhaps some increased facilities, on the part of government, to the procuring of such lymph from large towns, where alone it can be obtained in that state of purity and perfection which is essential to the safety of the individual in after life.

The experience which I have derived from private practice during the past year, has been, upon the whole, less favourable than that which my hospital records afford. It has occurred to me to witness four cases of small-pox after vaccination; three of them young ladies, and the fourth a young German from

Hamburgh. Two I attended in consultation with Mr. Tupper, one with Mr. Rose, and one with Mr. Bradley. In one of the cases only was the complaint modified: the other three had it severely, and the German died upon the eleventh day of the disease. All of them had been vaccinated, and (as it was supposed) carefully; and scars, sufficiently indicative of the process, were perceptible on the arms of each.

These cases, taken singly, would lead to a most undeserved distrust of vaccination. I am very well aware, indeed, that a few (perhaps eight or ten) cases of a similar kind might have been collected together; but the general voice of the public satisfactorily showed that the upper ranks of society suffered, during the past year, from small-pox much less than the lower; and, in spite of the importance attached to every case of failure, the balance was so decidedly in favour of the preventive powers of vaccination, that very few persons were met with whose faith was permanently shaken. The upper ranks of society still continue to be vaccinated as heretofore; and thus unite with the great mass of the British public in offering the strongest possible testimony to the value of the splendid discovery of Jenner.

8, *Upper John-street, Golden-square;*
*January* 12, 1826.

